# Minimally invasive (flapless) crown lengthening by erbium:YAG laser in aesthetic zone

**DOI:** 10.12688/f1000research.26008.3

**Published:** 2021-03-01

**Authors:** Saverio Capodiferro, Angela Tempesta, Luisa Limongelli, Giuseppe Barile, Daniela Di Venere, Massimo Corsalini

**Affiliations:** 1 Interdisciplinary Department of Medicine, University of Bari, Bari, Italy

**Keywords:** flap-less crown lengthening, Erbium:YAG laser; smile line

## Abstract

Crown lengthening is a surgical procedure aimed at exposure of a larger tooth surface by gingivectomy alone or with cortical bone remodelling for aesthetic purposes in the anterior zone of the maxilla or for reconstruction of teeth affected by subgingival caries. We report two cases of crown lengthening in the anterior maxilla for aesthetic purposes by gingival and bone re-contouring performed by erbium-doped yttrium aluminium garnet (erbium:YAG) laser. As highlighted in this report, the erbium:YAG laser-assisted crown lengthening is less invasive and also leads to faster clinical outcomes in contrast to the conventional surgical technique by scalpel incision, flap elevation and osteoplastic.

## Introduction

Several clinical situations may require dental crown lengthening (CL) such as irregular smile line, gummy smile, decayed or fractured teeth, worn out teeth by parafunction habits (e.g. bruxism) ^1, 2^. Regardless of aesthetic or functional purpose, the conventional technique of CL involves scalpel incision, flap elevation and bone remodeling by burns, with or without adjunctive gingivectomy, the latter essentially related to the gingival biotype ^3, 4^. Despite the excellent clinical outcome, the conventional surgical technique may be more invasive depending on the severity of the clinical situation as well patient’s general health condition (e.g. medically compromised patients or in therapy with anticoagulant drugs). Many alternatives techniques for CL have been reported in literature but it is generally accepted that the least invasive are the laser-assisted techniques ^5, 6^. Of these, the erbium:YAG laser has the advantage to work on both hard (bone) and soft tissues (gingiva) ^7^. We report on 2 cases treated by a mini-invasive erbium:YAG laser-assisted procedure (including gingiva and bone re-contouring) for CL in the anterior maxilla.

## Cases presentation

### Case 1

**Figure 1.**
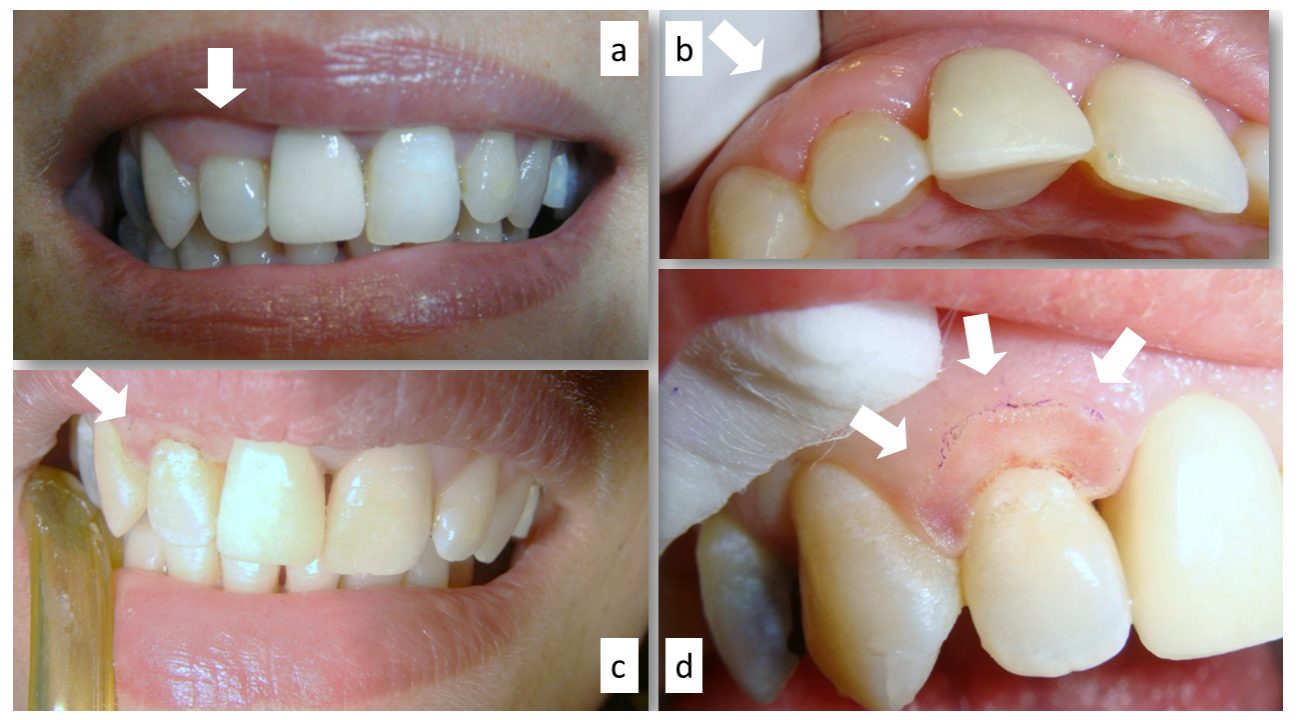
Alteration of the smile line related to the abundant gingiva of tooth 1.2 ( **a**, **b**); gingival remodelling by erbium-doped yttrium aluminium garnet (erbium:YAG) laser and its immediate clinical appearance ( **c**, **d**).

**Figure 2.**
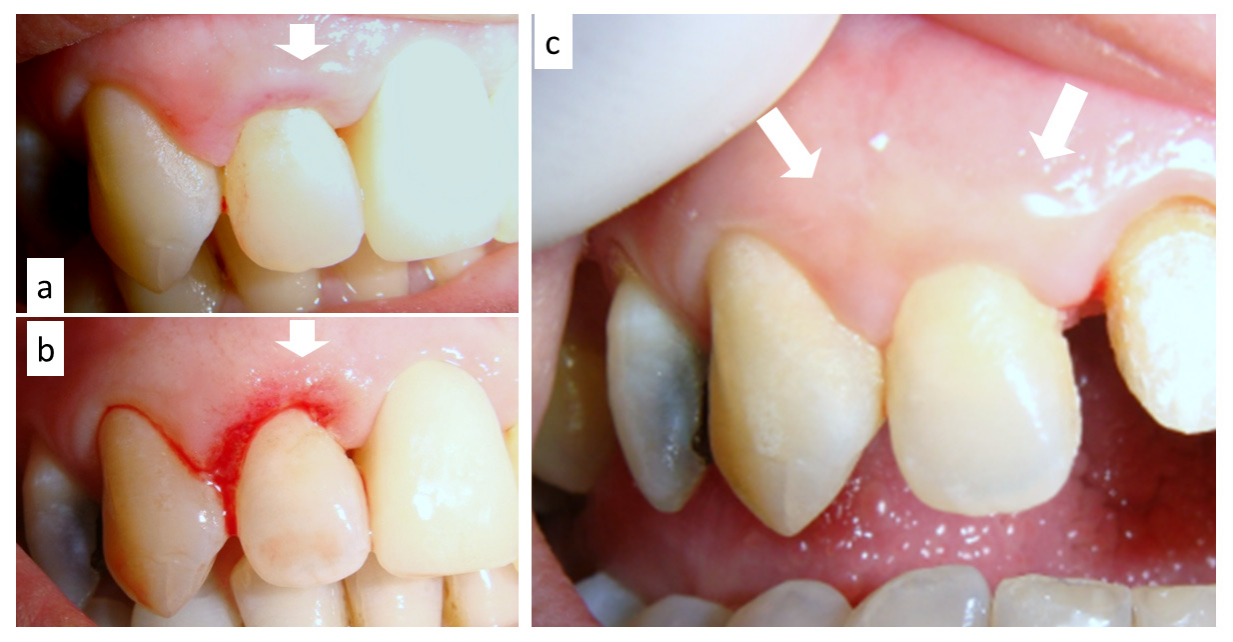
Second step after seven days ( **a**); flapless (through the gingival sulcus) bone re-contouring by erbium-doped yttrium aluminium garnet (erbium:YAG) laser ( **b**) and its clinical appearance after 12 days ( **c**).

The patient was a 53 y.o. Caucasian woman with an no relevant medical history who was unemployed at the time of presentation (March, 2015). She presented an abundant gingiva covering tooth 1.2 which she wished to remove for aesthetical purposes ( Figure 1a,b). Gingival remodeling and bone re-contouring by erbium:YAG laser was suggested. A small amount of anesthesia was injected locally (0.9 ml of mepivacaine cloridrate 2%, 1:100,000 epinephrine) after which the gingiva was remodeled by laser (Key Laser 3-Kavo s.r.l.-Italy) in de-focalized modality (not in contact free beam tip, 180 mJ/10 Hz, poor water emission) until the dental crown was sufficiently exposed according to the patient smile line ( Figure 1c,d). After one week ( Figure 2a), a second procedure was performed to re-contour the marginal bone by the same laser, using a surgical tip (optical prism scalpel-like tip of 01×10mm, 120 mJ/10 Hz, abundant water emission) in contact modality and through the gingival sulcus (flap-less); a light bleeding occurred during the procedure ( Figure 2b). The gingival margin was completely healed, and the smile line appeared significantly improved 12 days after surgery ( Figure 2c).

### Case 2

**Figure 3.**
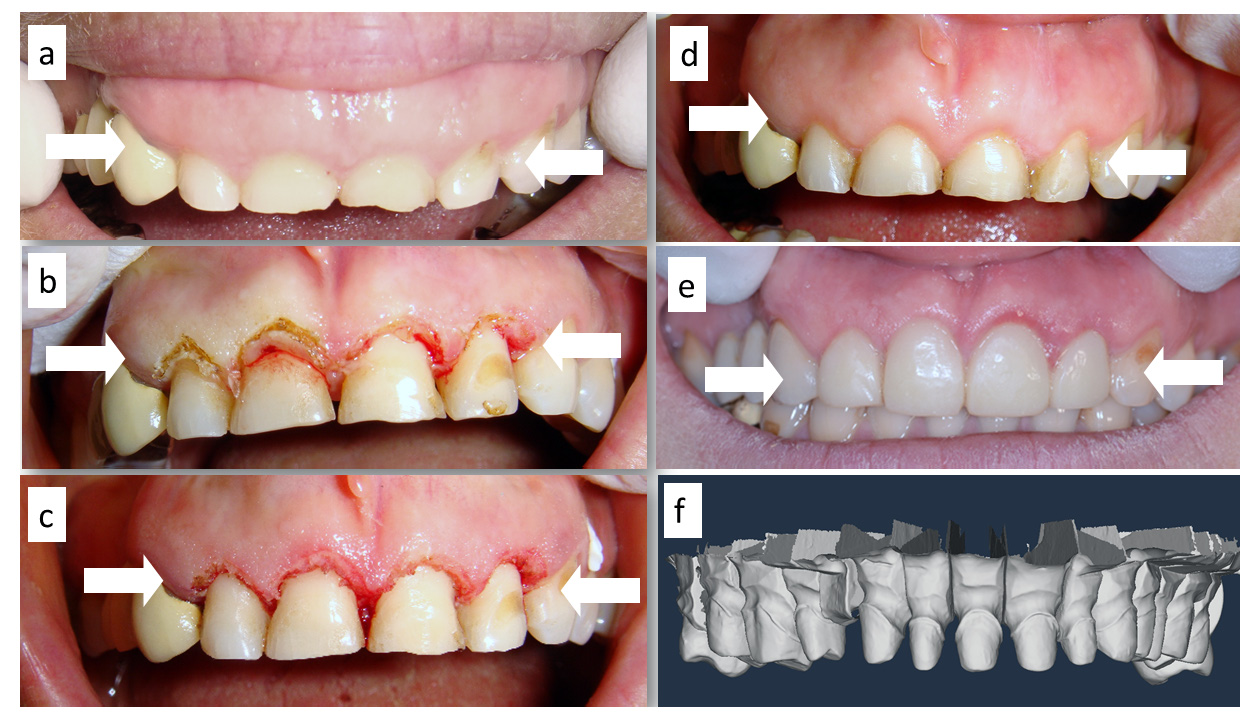
Severe abrasion of incisors due to bruxism ( **a**); erbium-doped yttrium aluminium garnet (erbium:YAG) laser-assisted gingivectomy ( **b**) and contextual flapless bone remodelling ( **c**); the clinical appearance after 14 days ( **d**), the teeth preparation as appearing on computer-aided design and the following prosthetic rehabilitation ( **e**, **f**).

This 47 y.o. Caucasian housewife who presented in April 2016 with severe abrasion of the anterior teeth related to bruxism over a long duration ( Figure 3a). Her medical history was un-remarkable. No pain and/or teeth hyper-sensibility were indicated by the patient, however, she was unhappy with her smile. A laser-assisted CL of the lateral and central incisors was planned to re-define a new marginal gingiva profile. After local injection of anesthesia, (1,8 ml of mepivacaine cloridrate 2%, 1:100,000 epinephrine), the marginal gingiva was careful recontoured by erbium:YAG laser (Key Laser 3-Kavo s.r.l.-Italy) (not in contact free beam tip, 180 mJ/10 Hz, poor water emission) till an adequate teeth exposure ( Figure 3b,c); subsequently, the cortical bone was-remodeled by a surgical tip (optical prism scalpel-like tip of 01×10mm, 160 mJ/10 Hz, abundant water emission) on both aspects of the maxilla through the gingival sulcus without flap elevation ( Figure 3d). After 14 days, gingival tissues appeared healed and teeth prepared for the following prosthetic restoration by cemented metal-ceramic crowns. ( Figure 3e,f).

## Discussion

Several medical devices have been proposed to make CL less invasive, including piezosurgery ^4, 8^. Several lasers such as diode, neodymium-doped yttrium aluminum garnet (Nd:YAG), potassium titanyl phosphate (KTP), CO _2_, Erbium, chromium-doped yttrium, scandium, gallium and garnet (Er,Cr:YSGG) and erbium:YAG are widely used for CL ^1, 2, 6, 9^. However, the main difference between these is their capability to work exclusively on soft or hard or both tissues ^3, 9, 10^. Diode, Nd:YAG, KTP and CO _2 _lasers may be useful when only gingival remodeling alone is necessary and this is essentially related to their surgical capabilities, especially contextual cuts and coagulation ^2, 9, 11, 12^. In fact, they are generally suggested for many surgical and non-surgical procedures in the oral cavity (frenectomy/frenulotomy, vestibuloplasty, mucosal biopsy, treatment of tooth hyper-sensibility, benign, potentially malignant and malignant lesions removal, surgical and not-surgical periodontal treatments including drug-related gingival overgrowth, photocoagulation of venous malformations, etc), but not for bone treatments ^12– 19^. When both gingival and bone remodelling is required, instead, the choice necessarily must fall on Er;Cr:YSGG or erbium:YAG lasers thanks to their selectivity for water, resulting in the capability to work by ablation on hard tissues as tooth and bone ^10, 11, 20, 21^. Therefore, such lasers can be used for dental cavity preparation, periodontal treatments, dentinal hypersensitivity, benign lesion removal, treatment of viral lesion of the oral mucosa and lip, gingival and/or bone remodelling or cutting ^7, 9, 10, 20^. In the reported cases, authors used an erbium:YAG laser both for soft and hard tissue treatment but with different tips and output energy parameters. The excellent clinical outcomes we described in terms of minimal invasiveness, lack of intra- and post-operative complications and pain, fast and predictable healing, are essentially related to the intrinsic proprieties of the erbium:YAG laser light and to the generally recognized gentle laser-oral tissues interaction ^10, 11, 20– 23^.

## Conclusion

The overall clinical benefits of the erbium:YAG laser allows flapless CL to be simplified, even in difficult cases. The total absence of laser-related thermal injuries to the oral hard and soft tissues leads to highly predictable clinical results, and this is important in the treatment of the anterior teeth for aesthetic purposes. However, a good knowledge of laser-tissue interaction principles, sufficient experience on laser use and, obviously, familiarity with the general and basic guidelines of oral/periodontal surgery are mandatory to achieve desirable clinical results.

## Consent

Written informed consent for publication of their clinical details and clinical images was obtained from the patient.

## Data availability

### Underlying data

All data underlying the results are available as part of the article and no additional source data are required.
