# Supplementary material for: Minimally invasive (flapless) crown lengthening by erbium:YAG laser in aesthetic zone
Source: F1000Res. 2021 Mar 1;9:1185. Originally published 2020 Sep 30. [Version 3] doi: 10.12688/f1000research.26008.3 (PMC7927205; doi:10.12688/f1000research.26008.3)
Supplement: Peer review history

# Peer review: Reviewer response for version 3

**DOI:** 10.5256/f1000research.54289.r80423

**Published:** 2021-03-02

**Authors:** Nasim Chiniforush

**Affiliations:** 1 Dental Implant Research Center, Dentistry Research Institute, Tehran University of Medical Sciences, Tehran, Iran

The article in this format is acceptable for indexing.

Are enough details provided of any physical examination and diagnostic tests, treatment given and outcomes?

Yes

Is the background of the cases’ history and progression described in sufficient detail?

Yes

Is sufficient discussion included of the importance of the findings and their relevance to future understanding of disease processes, diagnosis or treatment?

Yes

Is the conclusion balanced and justified on the basis of the findings?

Yes

Reviewer Expertise:

photodynamic therapy, laser surgery, photobiomodulation, hard tissue surgery, soft tissue surgery

I confirm that I have read this submission and believe that I have an appropriate level of expertise to confirm that it is of an acceptable scientific standard.

# Peer review: Reviewer response for version 2

**DOI:** 10.5256/f1000research.54007.r77858

**Published:** 2021-01-26

**Authors:** Nasim Chiniforush

**Affiliations:** 1 Dental Implant Research Center, Dentistry Research Institute, Tehran University of Medical Sciences, Tehran, Iran

- The unit for energy should be mJ not MJ. 
- What does poor emission mean? Give exact amount of water irrigation.
- The country of laser device should be added.
- It's better to add this citations:
  - Fekrazad R, Moharrami M, Chiniforush N. The Esthetic Crown Lengthening by Er;Cr:YSGG laser: A Case Series. J Lasers Med Sci. 2018 Fall;9(4):283-287. doi: 10.15171/jlms.2018.50. Epub 2018 Sep 17. PMID: 31119024; PMCID: PMC6499554. ^1^
  - Flax HD, Radz GM. Closed-flap laser-assisted esthetic dentistry using Er:YSGG technology. Compend Contin Educ Dent. 2004 Aug;25(8):622, 626, 628-30 passim. PMID: 15456127. ^2^

Are enough details provided of any physical examination and diagnostic tests, treatment given and outcomes?

Yes

Is the background of the cases’ history and progression described in sufficient detail?

Yes

Is sufficient discussion included of the importance of the findings and their relevance to future understanding of disease processes, diagnosis or treatment?

Yes

Is the conclusion balanced and justified on the basis of the findings?

Yes

Reviewer Expertise:

photodynamic therapy, laser surgery, photobiomodulation, hard tissue surgery, soft tissue surgery

I confirm that I have read this submission and believe that I have an appropriate level of expertise to confirm that it is of an acceptable scientific standard, however I have significant reservations, as outlined above.

## References

[1] : The Esthetic Crown Lengthening by Er;Cr:YSGG laser: A Case Series. *Journal of Lasers in Medical Sciences*.2018;9(4) : 10.15171/jlms.2018.50 283-287 10.15171/jlms.2018.50 PMC649955431119024

[2] : Closed-flap laser-assisted esthetic dentistry using Er:YSGG technology. *Compend Contin Educ Dent*.2004;25(8) :622, 626, 628-30 passim 15456127

# Response

**Published:** 2021-01-27

**Authors:** saverio capodiferro

**Affiliations:** University of Bari, Italy

Dear Reviewer, thank you for your suggestions. Changes (MJ with mJ, adding of references and Country of laser device as suggested) will be performed accordingly in the next version. With regard to the water emission, I'm very sorry but it is impossible to identify correctly the quantity as limitation of the laser device.

 Thank you again.

# Peer review: Reviewer response for version 1

**DOI:** 10.5256/f1000research.28703.r72300

**Published:** 2020-12-03

**Authors:** Nasim Chiniforush

**Affiliations:** 1 Dental Implant Research Center, Dentistry Research Institute, Tehran University of Medical Sciences, Tehran, Iran

- In two cases please add pulse duration of laser device you used for each purpose and laser tip diameter and length.
- You can add some more information about the advantages of erbium lasers.
- It's better to add case report in title of your manuscript.
- This article after minor revision can be acceptable for indexing.

Are enough details provided of any physical examination and diagnostic tests, treatment given and outcomes?

Yes

Is the background of the cases’ history and progression described in sufficient detail?

Yes

Is sufficient discussion included of the importance of the findings and their relevance to future understanding of disease processes, diagnosis or treatment?

Yes

Is the conclusion balanced and justified on the basis of the findings?

Yes

Reviewer Expertise:

photodynamic therapy, laser surgery, photobiomodulation, hard tissue surgery, soft tissue surgery

I confirm that I have read this submission and believe that I have an appropriate level of expertise to confirm that it is of an acceptable scientific standard, however I have significant reservations, as outlined above.

# Response

**Published:** 2021-01-15

**Authors:** saverio capodiferro

**Affiliations:** University of Bari, Italy

Thank you very much. Changes have been made accordingly.

# Peer review: Reviewer response for version 1

**DOI:** 10.5256/f1000research.28703.r72302

**Published:** 2020-11-17

**Authors:** Rada T. Kazakova

**Affiliations:** 1 Department of Prosthetic Dentistry, Faculty of Dental Medicine, Medical University of Plovdiv, Plovdiv, Bulgaria

Dr. Saverio Capodiferro *et al.*’s article reports two cases of a crown lengthening procedure with an Er:YAG laser. This procedure aims at exposure of a larger tooth surface usually on the anterior maxillary region by gingivectomy alone (soft tissue crown lengthening), or gingivectomy and cortical bone remodeling (hard tissue crown lengthening). The advantages of the Er:YAG method, compared to the classical scalpel one, is that it leads to a predictable outcome and a faster healing

 The background of the cases’ history and progression described is in sufficient detail. The authors have introduced us to the patients, their age, history of the disease and the reason why they sought for the crown lengthening procedure in particular. There is enough data provided of the examinations and diagnostic tests, treatment given and outcomes. The authors described in detail what the gingival level was, and illustrated the gingival overgrowth, the steps and the clinical outcome with excellent photos, including a CAD/CAM scan. They also added photos after the procedures and the results after the final restorations. The discussion is sufficient and it accentuates on the importance of the outcome and the better and faster healing of the Er:YAG laser crown lengthening, compared to the classical method with the scalpel. It gives much details on the wide areas of use of the different lasers, as well as a comparison between them – their advantages and disadvantages, as well as their indications and contraindications. The conclusion is balanced and summarizes the findings. One of the most important advantage of the Er:YAG laser method described is the better healing and the predictable results, which is mentioned in the conclusion. My only small recommendations for them, which are not obligatory, are the following: to change ‘a small anesthesia’ to ‘a little bit of anesthesia’ and to describe what type of anesthesia they used (e.g. terminal, intra-ligamental, etc.); ‘light bleeding’ – should be written without ‘a’; it would be useful if they could provide the readers with some more information and the precise specification of the laser, laser mode and scalpel tip used; ‘her medical history was un-remarkable’ is a statement that I could not quite understand, maybe the readers need some more clarity, as well.

 I congratulate the team with the good work, the efforts and the wonderful outcomes and wish them to more and more success in the future. The article is interesting and well-described. I will definitely vote ‘approved’ for this article.

Are enough details provided of any physical examination and diagnostic tests, treatment given and outcomes?

Yes

Is the background of the cases’ history and progression described in sufficient detail?

Yes

Is sufficient discussion included of the importance of the findings and their relevance to future understanding of disease processes, diagnosis or treatment?

Yes

Is the conclusion balanced and justified on the basis of the findings?

Yes

Reviewer Expertise:

Laser dentistry

I confirm that I have read this submission and believe that I have an appropriate level of expertise to confirm that it is of an acceptable scientific standard.
